# The mesenchymal circulating tumor cells as biomarker for prognosis prediction and supervision in hepatocellular carcinoma

**DOI:** 10.1007/s00432-022-04526-9

**Published:** 2023-01-12

**Authors:** Lina Zhao, Zhifa Zheng, Yunhe Liu, Fei Liu, Xiaoxin Li, Zhihong Wu

**Affiliations:** 1 Medical Research Center, Peking Union Medical College Hospital, Peking Union Medical College and Chinese Academy of Medical Sciences, Beijing, 100730 China; 2 Key Laboratory of Big Data for Spinal Deformities, Chinese Academy of Medical Sciences, Beijing, 100730 China; 3 State Key Laboratory of Complex Severe and Rare Diseases, Peking Union Medical College Hospital, Peking Union Medical College and Chinese Academy of Medical Sciences, Beijing, 100730 China; 4 Department of Hepatobiliary Surgery, National Cancer Center/National Clinical Research Center for Cancer/Cancer Hospital, Chinese Academy of Medical Sciences and Peking Union Medical College, Beijing, China

**Keywords:** Circulating tumor cells, Epithelial–mesenchymal transition, Hepatocellular carcinoma, Prognosis prediction

## Abstract

**Purpose:**

Hepatocellular carcinoma (HCC) is one of the most common cancers and a leading cause of death worldwide. Accurate prognosis prediction tools are urgently needed. While the use of circulating tumor cells (CTCs) as prognostic prediction tool has a clear potential.

**Methods:**

We established a comprehensive, negative enrichment-based strategy for CTCs analysis in patients with HCC, involving identification of epithelial CTCs (E-CTCs) and mesenchymal CTCs (M-CTCs) through specific biomarker. This strategy was performed in 127 HCC cases, 21 nonmalignant liver disease (NMLD) patients and 42 health control to analyze the relevance between CTCs and tumor recurrence.

**Results:**

The total CTC number and M-CTC percent were positively correlated with tumor malignancy and high recurrence risk. Individually, preoperative total CTC number and M-CTC percent could robustly distinguish relapse cases from those with no relapse, with sensitivity of 80.95% and 90.48%, specificity of 74.12% and 84.71%, respectively. Levels of preoperative total CTC number and M-CTC percent can both be regarded as independent risk factors for HCC with early recurrence (*P* = 0.0053, *P* < 0.0001), and are both significantly correlated with worse recurrence-free survival (RFS) (log rank *P* < 0.0001; HR 7.78, 95% CI = 3.59–16.87; log rank *P* < 0.0001; HR 24.4, 95% CI = 8.67–68.77). The levels of total CTC number and M-CTC number had higher effectiveness than alpha fetal protein (AFP) in HCC longitudinal supervision (77.78% vs 88.89% vs 22.22%).

**Conclusion:**

Preoperative and postoperative CTCs with higher effectiveness than AFP in prognosis prediction and recurrence supervision, indicating that CTCs could work as the biomarker for HCC clinical management.

**Supplementary Information:**

The online version contains supplementary material available at 10.1007/s00432-022-04526-9.

## Introduction

Hepatocellular carcinoma (HCC) was responsible for an estimated 905,000 new incidence and nearly 830,000 mortalities in 2020 world-wide (Sung et al. 2021). It ranks as the fourth most common malignant cancer and the third most frequent cause of cancer related death in China (Chen et al. 2016). The etiology of HCC has a certain correlation with population and geographical demographics. Approximately 80% of patients with HCC in China are associated with hepatitis B virus (HBV) infection (El-Serag and Rudolph 2007). As a crucial environmental risk factor, uncontrolled dietary aflatoxin exposure is pervasive not only in China, but also in sub-Saharan Africa and Southeast Asia (Liu and Wu 2010). Other known risk factors of HCC include hepatitis C virus (HCV) infection, diabetes, obesity, smoking, alcohol consumption, nonalcoholic fatty liver disease, hereditary diseases, and being male (Bosetti et al. 2014; Kamangar et al. 2006).

Traditional imaging methods, such as computer tomography (CT), magnetic resonance imaging (MRI) and positron emission tomography computed tomography (PET-CT) are inefficient in evaluating disease malignancy and monitoring the minimal residual disease (MRD) in some cases (Schraml et al. 2015). Currently, alpha fetal protein (AFP) is the only approved serum biomarker for HCC, however, the low sensitivity of tests for AFP limits its application (Johnson 2001). The diagnostic and prognostic efficacy of other serum protein biomarkers, such as AFP-L3 and Des-γ-carboxy (DCP), are only slightly higher than that of AFP (Wang et al. 2005; Wei et al. 2018). At present, the existing clinical staging systems, such as Okudo staging, Barcelona Clinic Liver Cancer (BCLC) staging and American Association of Cancer (AJCC) staging, have limited effect on HCC staging and prognosis estimation, and cannot be used solely based on preoperative clinical data (Huang et al. 2005; Ishizawa et al. 2008).

Therefore, there is a great need for non-invasive biomarkers which can efficiently classify the malignant degree of HCC and accurately predict the chance of postoperative recurrence. Liquid biopsy, measuring tumor byproducts such as circulating tumor cells (CTCs), cell­-free tumor DNA (cfDNA) and so on, is an innovative testing method which can supplement the flaws of measuring only current serum biomarkers (Palmirotta et al. 2018). The tumor related information revealed by liquid biopsy has potential for early diagnosis, treatment response monitoring and presage tumor recurrence. As an important measured output of liquid biopsy, CTCs are tumor cells with high colonization ability and metastatic potential in the peripheral blood. There is a clear correlation between the number and phenotype of CTCs and the progression of tumor, which conducted to a valid window to understand the nature of tumor lesions (Amin et al. 2017). The concept that CTCs can be used to monitor tumor progression, metastasis, recurrence and prognosis is widely recognized (Tjensvoll et al. 2014, Alix-Panabieres and Pantel 2014).

After invading into the circulation system, CTCs can evade recognition and elimination by the immune system, then spread to distant organs and form metastatic lesions (Cho et al. 2012). The epithelial–mesenchymal transition (EMT) and its reverse process, the mesenchymal–epithelial transition (MET) are believed to play key roles in this process. EMT is a complicated process that endows epithelial cells with mesenchymal cell traits and enhanced metastatic and invasive capability (Vardas et al. 2021). During EMT, the expression of epithelial markers, such as the epithelial cell adhesion molecule (EpCAM) and cytokeratins (CK), will be downregulated, while expression of mesenchymal markers, such as vimentin and twist, will be upregulated (Kalluri 2009). The change between epithelial and mesenchymal states is plastic; CTCs may go through dynamic processes of EMT and MET, and acquire various phenotypes on the spectrum of epithelial to mesenchymal. CTCs with mesenchymal markers have been found in multiple cancers, including breast, lung, prostate, and liver cancer (Khoshbakht et al. 2021; Jiang et al. 2021).

The combination of the epithelial and mesenchymal markers has potential as a relatively more sensitive and accurate CTCs detection strategy compared to current methods. According to the previous researches, vimentin can serve as an identification biomarker of CTCs undergoing EMT (Satelli et al. 2015; Li et al. 2018). To detect both the epithelial and mesenchymal CTCs in peripheral blood of HCC patients, the antibody combination of CK, vimentin and CD45 was applied in this study. After excluding white blood cells (WBC) according to the expression of CD45, we classified the CTCs based on expression of vimentin and CK as either epithelial CTCs (E-CTCs), mesenchymal CTCs (M-CTCs) and epithelial–mesenchymal CTCs (EM-CTCs). We then investigated the correlation between clinical/pathological features and different phenotype of CTCs, and evaluated its potential prognostic value. It turned out that the M-CTC percent was a more effective indicator than total CTC number in HCC recurrence prediction. Also, total CTC number > 10/7.5 ml and M-CTC percent of > 23.33% were correlated with a worse recurrence free survival (RFS) rate, also could be used to independently predict HCC early recurrence. In addition, CTCs could work as an effective recurrence indicator in HCC supervision, especially for the AFP negative patients.

## Methods

### Patient enrollment and follow-up

From March 2017 to September 2019, a total of 127 HCC patients from the Cancer Hospital of Chinese Academy of Medical Sciences were included in the study group. All patients were diagnosed with HCC by pathologic and fine-needle aspiration biopsy. Another 42 healthy controls and 21 patients with nonmalignant liver disease (NMLD) (adenoma: *N* = 1; liver cirrhosis without HCC: *N* = 15; focal nodular hyperplasia: *N* = 2; hepatic hemangioma: *N* = 3) were enrolled. The tumor stages of HCC patients were defined according to the Barcelona Clinic Liver Cancer (BCLC) and American Joint Committee on Cancer (AJCC) staging systems. Tumor differentiation was stratified based on the Edmondson grading system. Clinical information such as age, sex, hepatitis virus infection status and serum tumor biomarkers were collected. In addition, the biochemical examination, imaging data and pathological results of HCC patients were collected. The clinical characteristics of HCC patients, healthy controls and NMLD patients were presented in Table S1, Table S2 and Table S3, respectively. After operation, patients were followed up every 3 months during the first postoperative year, and every 6 months afterwards. Patients suspected to have recurrence and metastasis were monitored by serum AFP, abdomen ultrasonography, CT, MRI and/or PET CT. Follow-up was terminated on September 30, 2021, and recurrence-free survival (RFS) was defined as the time interval between the operation and recurrence or the endpoint of follow-up. The study was approved by the ethics review committee of Cancer Hospital of Chinese Academy of Medical Sciences (ID: NCC3094). Informed consent was obtained from participants in accordance with respective committee regulations and the study was performed in accordance with the guidelines of the Declaration of Helsinki.

### Establishment of recurrence risk index

A high recurrence risk index was established based on methods in previous studies characterizing the pathological character of tumors. The criteria included: tumor number > 1; tumor diameter > 5 cm; macroscopic vascular invasion (tumor thrombus); pathologic microvascular invasion (MVI); satellite nodules; the T stages of AJCC was 2, 3 or 4; histopathological diagnosis was poorly differentiated or undifferentiated, the index over 0 was defined as high recurrence risk patient (Xu et al. 2019; Shah et al. 2007).

### Blood sample collection and CTCs enrichment

The first 2 ml blood was discarded to avoid contamination from epithelial cells of the skin. Then, 8–10 mL blood sample was collected in Streck tubes (STRECK, USA) and transferred to laboratory. Then, 7.5 ml blood sample was precisely added into centrifuge tube with pipette, then it was diluted with equal volume of phosphate buffered saline (PBS) buffer under the aseptic condition. 3 ml of Histopaque-1077 reagent (Sigma–Aldrich, USA) added into the bottom of Leucosep^®^ tube (Greiner Bio-One, Germany) and balanced to room temperature. Then, the diluted blood sample was added into the upper channel of Leucosep^®^ tube and centrifuged at 1000 g/10 min at room temperature to separate peripheral blood mononuclear cells (PBMC). The buffer coat containing the PBMC was transferred to a new 15 mL tube, and cells were gently resuspended with 10 mL washing buffer (0.5% Bovine Serum Albumin (BSA), 137 mM NaCl, 2.7 mM KCl, 10 mM Na2HPO4, 2 mM KH2PO4, 1 mM EDTA, pH 7.4), and centrifuge at 300*g* at 4 °C for 10 min, the supernatant was discarded and this was repeated one more time to obtain nucleated cells. Then, anti-human CD45 immunomagnetic beads (Miltenyi Biotec, Germany) were added in equal proportion (20 ul/10^7^ WBC) and incubated at 4 °C for 15 min. The supernatant was removed, 500 uL buffer was added to resuspend the pellet, then the cell suspension was transferred to the LS column (Miltenyi Biotec, Germany) under a strong magnetic field, eluted and nucleated cells not bound to the CD45 magnetic beads (including CTCs) were collected. The supernatant was transferred to a new tube centrifuged at 1000*g*/10 min and subsequently spotted on a glass slide.

### CTCs identification

The CTC droplet slides were blocked with PBS containing 2% BSA for 30 min, and then anti Vimentin-AlexaFluor 647 (1:200) (Cell Signaling Technology, CAT: 98565), anti-CK8/18/19-FITC (1:100) (Miltenyi Biotec, CAT: 130-080-101) and anti-CD45- PE (1:200) (Miltenyi Biotec, CAT: 130-045-801) diluted in 2% BSA were added and incubated at room temperature for 1 h. Then, the slides were washed three times with PBS, 3 min/time to remove unbound antibody. The CTC slides were mounted with the nuclear dye 4′,6-diamidino-2-phenylindole (DAPI) (Sigma–Aldrich, CAT: D9564) and observed under a fluorescence microscope (Nikon, Japan). The epithelial CTCs (E-CTCs) were defined as PanCK (+), vimentin (−), CD45 (−) and DAPI (+) cells, the mesenchymal CTCs (M-CTCs) were defined as PanCK (−), vimentin (+), CD45 (−) and DAPI (+) cells, the epithelial–mesenchymal CTCs (EM-CTCs) were defined as PanCK (+), vimentin(+), CD45 (−) and DAPI (+) cells, whereas white blood cells (WBCs) were defined as PanCK (−), vimentin (−), CD45 (+) and DAPI (+) cells, respectively. Total CTC number were calculated as the sum of E-CTCs, M-CTCs and EM-CTCs of each patient. For some patients whose blood collection does not reach 7.5 ml, the final number of CTC is calculated by the following formula: number of CTC/volume of blood used in the experiment * 7.5 ml.

### Statistical analysis

The statistical analysis was conducted through GraphPad Prism version 9.0 software (GraphPad Software, USA) and R software (Version 3.6.1, R Foundation for Statistical computing, Austria). All measured data are presented as the mean ± standard deviation (mean ± SD). The Mann–Whitney rank test was used to compare the differences between two groups, and the analysis of variance (ANOVA) was used to compare the differences among the more than two groups by multiple comparisons test. The “pROC” package of R was performed to establish the receiver operating characteristic (ROC) curve to evaluate the diagnostic and prognostic value of different subtype of CTCs. The “survival” and “survminer” were used to conduct univariate and multivariate analysis of Cox regression model. The continuous parameters and the total CTC number and M-CTC percent were dichotomized for RFS before the log-rank test by optimal cutoff values determined based on the ROC curve. All statistical tests were two-tailed and considered significant if *P* < 0.05 (*P < 0.05, **P < 0.01, ***P < 0.001 and ****P < 0.0001).

## Results

### Patient characteristics

A total of 127 HCC patients were enrolled in this study. The clinical and pathological characteristics of the 127 recruited patients are summarized in Table S1. The cohort includes 106 male patients (83.46%) and 21 female patients (16.53%), with a median age of 55 (range 28–77). 39 patients (30.71%) had received adjuvant therapy before hepatectomy. 87.40% (111/127) of patients were hepatitis B virus (HBV) positive and 8.66% (11/127) were hepatitis C virus (HCV) positive. In addition, 116 patients (91.34%) presented with cirrhosis. The cohort was largely composed of early stage HCC patients, with 66.93% (85/127), 11.02% (14/127) and 22.05% (28/127) of patients in BCLC stage A, B and C, respectively, and 42.06% (53/127), 36.51% (46/127), 20.63% (26/127) and 0.79% (1/127), in AJCC stages I, II, III, and IV, respectively (Table S1). In addition, 42 healthy control subjects, including 29 males (69.05%) and 13 females (30.95%) with a median age of 54 (range 23–76), and 21 patients with nonmalignant liver disease (NMLD), including 18 males (85.71%) and 3 females (14.29%) with a median age of 52 (range 32–72), were recruited at the same time. The clinical characteristics are summarized in Tables S2 and S3, respectively.

### Identification of different EMT phenotypes of CTCs

**Fig. 1:**
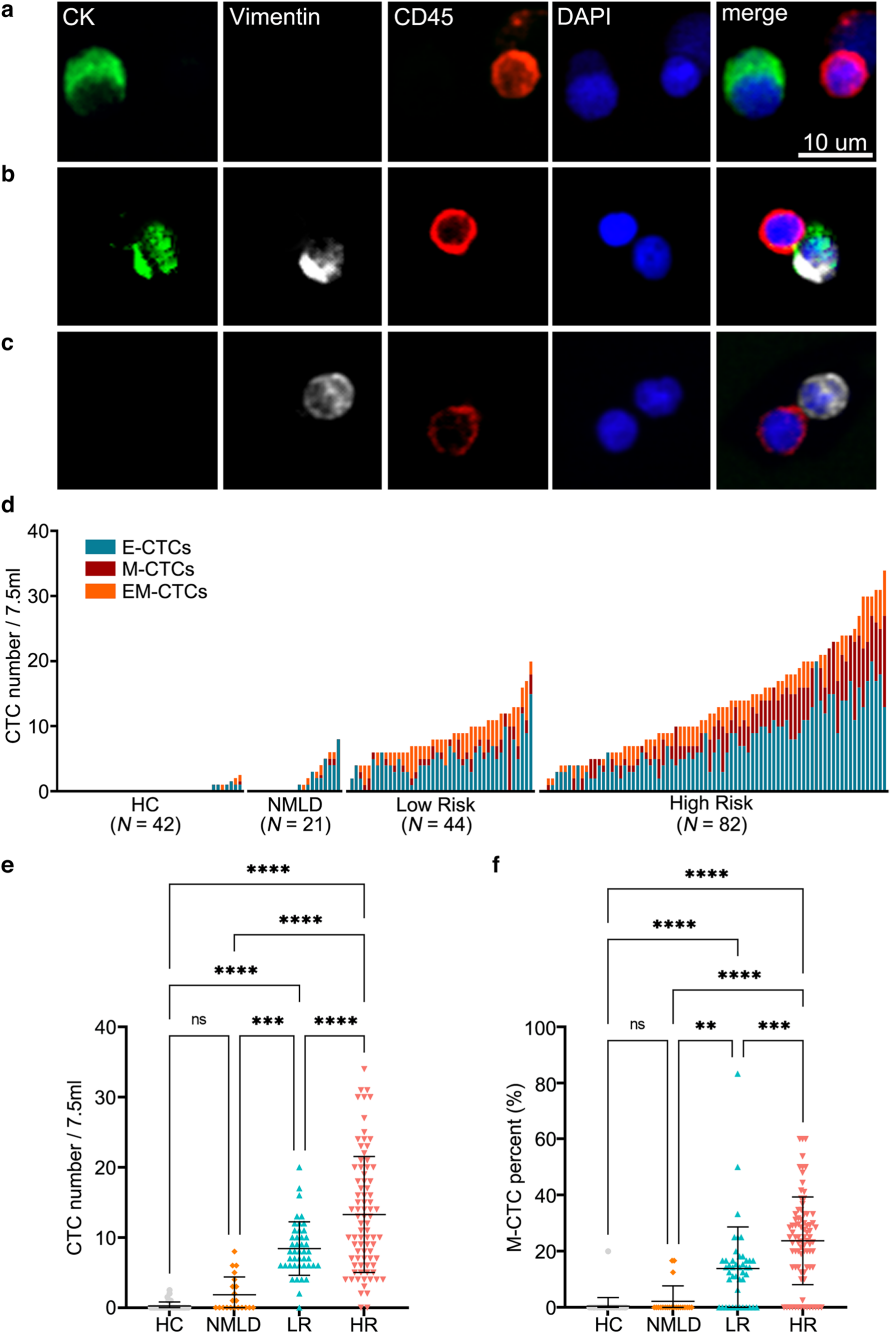
The distribution of EMT-related subtype of CTCs in different patient groups. **a** Epithelial CTCs (E-CTCs) were identified as Pan CK (+), vimentin (−), CD45 (−) and DAPI (+) cells at 400× magnification. **b** Epithelial-mesenchymal CTCs (EM-CTCs) were identified as Pan CK (+), vimentin (+), CD45 (−) and DAPI (+) cells at 400× magnification. (**c**) Mesenchymal CTCs (M-CTCs) were identified as Pan CK (−), vimentin (+), CD45 (−) and DAPI (+) cells at ×400 magnification. The scale bar indicated 10 μm in (**a**–**c**). (**d**) Counts for E-CTCs, EM-CTCs and M-CTCs for all participants enrolled in the study (*N* = 189). HCC patients are divided into groups based on the high risk index and sorted within groups based on total CTC count. **e**, **f** Preoperative total CTC number and M-CTC percent in cases with high recurrence risk (HR) versus low risk (LR), NMLD and HC group (one way ANOVA test). *CTCs* circulating tumor cells, *Pan CK* pan-cytokeratin, *DAPI* 4′,6-diamidino-2-phenylindole, *HC* health control, *NMLD* nonmalignant liver disease

EMT-related CTCs in the preoperative peripheral blood of 127 HCC patients after negative enrichment were identified using Pan-CK, vimentin and CD45 antibodies. Sample images of E-CTCs, M-CTCs, EM-CTCs and a few residual CD45^+^ white blood cells (WBC) are shown in Fig. 1a–c. In all HCC patients, the detection rate of E-CTCs (92.91%, 118/127) was slightly higher than that of M-CTCs (79.53%, 101/127) and EM-CTCs (81.89%, 104/127). The median numbers of E-CTCs, M-CTCs and EM-CTCs in 7.5 mL of preoperative peripheral blood from HCC patients in our cohort were 5 (range 0–20), 2 (range 0–14) and 2 (range 0–8), respectively.

### Correlation between tumor malignancy and different subtypes of EMT-related CTCs

A high recurrence risk index was established based on previous studies, using pathological characteristics of the tumor. A patient with an index over 0 was defined as a high recurrence risk patient (Xu et al. 2019; Shah et al. 2007). Among recruited HCC patients, 34.65% (44/127) were low risk (high recurrence risk index = 0), and 64.57% (82/127) were high risk (high recurrence risk index > 0). In the healthy control group, NMLD group, low risk, and high risk group, the median numbers of total CTCs (the sum of E-CTCs, M-CTCs and EM-CTCs) were 0/7.5 ml (range 0–2.5/7.5 ml), 0/7.5 ml (range 0–8/7.5 ml), 8/7.5 ml (range 0–20/7.5 ml) and 12/7.5 ml (range 0–34/7.5 ml), respectively (Fig. 1d). More total CTCs were found in HCC patients compared to healthy controls or NMLD patients (low risk: *P* < 0.0001, *P* = 0.0002; high risk: *P* < 0.0001, *P* < 0.0001), meanwhile, more CTCs were found in high risk HCC patients compare with low risk HCC patients (*P* < 0.0001) (Fig. 1e). Similarly, the percent of M-CTC was found much higher in HCC patients compared to NMLD patients or healthy controls (low risk: *P* < 0.0001, *P* = 0.0039; high risk: *P* < 0.0001, *P* < 0.0001), the percent of M-CTC in high risk HCC patients were much higher than that in low risk HCC patients (*P* = 0.0003) (Fig. 1f).

**Fig. 2:**
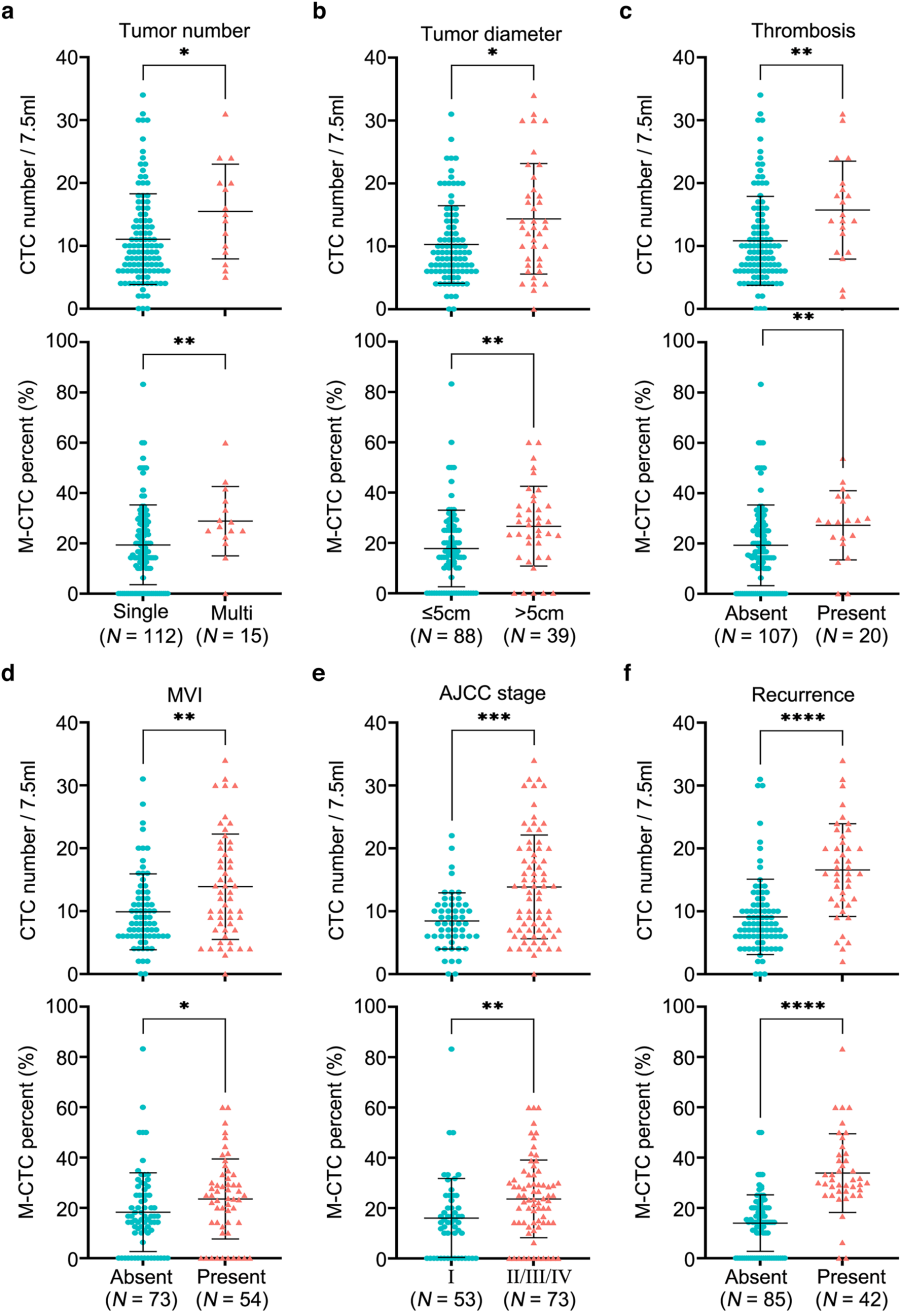
Correlation between total CTC number or M-CTC percent and HCC clinical parameters. **a**–**f** Scatter plots (upper panel) show the comparison of preoperative total CTC number under different clinical stratification criteria including tumor number, tumor diameter, thrombosis, MVI, AJCC stage, and recurrence state. Scatter plots (lower panel) showing the comparison of preoperative M-CTC percent under different clinical stratification criteria including tumor number, tumor diameter, thrombosis, MVI, AJCC stage, and recurrence state. (Mann Whitney *t* test, two-tailed). *CTCs* circulating tumor cells, *MVI* microvascular invasion, *AJCC* American Joint Committee on Cancer

Next, we evaluated the correlation between the levels of total CTC number and M-CTC percent with clinical parameters related to the poor prognosis of HCC. In this analysis, the amount of total CTCs in the peripheral blood was associated with the tumor number (*P* = 0.0233), tumor size as measured by the largest diameter (*P* = 0.0115), thrombosis (*P* = 0.0035), microvascular invasion (MVI, *P* = 0.0076), AJCC stage (*P* = 0.0003), and BCLC stage (*P* = 0.0007), indicating that total CTC levels are associated with HCC tumor burden and could help assess HCC progression (Fig. 2*,* Table 1).

**Table 1:** Correlation between clinical parameters and total CTC number or mesenchymal CTC (M-CTC) percent in HCC patients

Characteristic	Number (%)	Total CTC number (Mean ± SD)	*P*	M-CTC percent (%) (Mean ± SD)	*P*
Sex (*N* = 127)			0.6020		0.5159
Female	21 (16.53)	11.00 ± 6.87		14.29 ± 14.59	
Male	106 (83.46)	9.50 ± 7.42		20.00 ± 16.08	
Age, years (*N* = 127)			0.4691		0.8054
≤ 60	88 (69.29)	10.00 ± 7.40		20.00 ± 16.03	
> 60	39 (30.71)	8.00 ± 7.14		20.00 ± 15.45	
AFP (*N* = 127)			0.0374*		0.4803
≤ 20	72 (56.69)	9.00 ± 6.17		16.67 ± 15.65	
> 20	55 (43.31)	11.00 ± 7.89		20.00 ± 15.95	
HBV infection (*N* = 127)			0.0688		0.2300
Absent	16 (12.60)	7.50 ± 8.43		13.39 ± 15.85	
Present	111 (8.74)	10.00 ± 7.11		20.00 ± 15.76	
HCV infection (*N* = 127)			0.2527		0.7678
Absent	116 (91.34)	10.00 ± 7.24		20.00 ± 16.22	
Present	11 (8.66)	11.00 ± 7.89		20.00 ± 11.27	
Cirrhosis (*N* = 127)			0.0085**		0.4447
Absent	11 (8.66)	4.00 ± 6.36		14.29 ± 17.27	
Present	116 (91.34)	10.00 ± 7.30		20.00 ± 15.67	
Differentiation grade (*N* = 127)			0.5986		0.0710
Well	4 (3.15)	8.50 ± 2.69		15.75 ± 11.83	
Well-moderately	2 (1.57)	12.50 ± 10.50		17.39 ± 17.39	
Moderately	79 (62.20)	11.46 ± 7.08		18.97 ± 15.29	
Moderately-poorly	19 (14.96)	10.37 ± 5.95		17.84 ± 12.32	
Poorly	23 (18.11)	13.48 ± 8.84		29.19 ± 17.78	
AJCC stage (*N* = 126)			< 0.0001****		0.0335*
Ι	53 (42.06)	8.45 ± 4.41		16.08 ± 15.57	
II	46 (36.51)	12.41 ± 8.03		21.84 ± 15.17	
III	26 (20.63)	15.92 ± 7.74		26.66 ± 15.54	
IV	1 (0.79)	27 ± 0		29.63 ± 0	
Child Pugh class (*N* = 127)			0.1070		0.1332
A	118 (92.91)	11.20 ± 7.19		19.78 ± 15.64	
B	8 (6.30)	16.38 ± 7.84		29.32 ± 16.01	
C	1 (0.78)	18.00 ± 0		38.89 ± 0	
BCLC stage (*N* = 127)			0.0007***		0.0614
A	85 (66.93)	9.88 ± 6.31		18.34 ± 16.33	
B	14 (11.02)	15.93 ± 7.42		27.88 ± 12.73	
C	28 (22.05)	14.57 ± 8.27		23.48 ± 14.17	
Recurrence (*N* = 127)			< 0.0001****		< 0.0001****
Absent	85 (66.93)	8.00 ± 5.97		14.29 ± 11.20	
Present	42 (33.07)	16.00 ± 7.30		18.89 ± 15.52	

*HCC* hepatocellular carcinoma, *MVI* microvascular invasion, *AFP* alpha-fetoprotein, *CTC* circulating tumor cell, *HBV* hepatitis B virus, *HCV* hepatitis C virus, *AJCC* American Joint Committee on Cancer, *BCLC* Barcelona clinic liver cancer stage

*, **, ***, ****denote a *P* value of < 0.05, < 0.01, <0.001 and < 0.0001, respectively

Similarly, higher M-CTC percent was found in patients with multi tumor nodus (*P* = 0.0099), larger tumor sizes (*P* = 0.0011), thrombosis present (*P* = 0.0089), MVI present (*P* = 0.0231), higher AJCC stage (*P* = 0.0016), which suggests that relative M-CTC levels can assess disease progression of HCC patients in addition to total CTC number (Fig. 2*,* Table 1). In addition, neither total CTC number nor M-CTC percent were correlated with other clinicopathological features (sex: *P* = 0.6020, *P* = 0.5159; age: *P* = 0.4691, *P* = 0.8054; HBV infection: *P* = 0.0688, *P* = 0.2300; HCV infection: *P* = 0.2527, *P* = 0.7678; differentiation: *P* = 0.5986, *P* = 0.0710) (Table 1).

### M-CTCs could work as a more precise prognostic biomarkers in HCC patients

Based on stratification of the high risk index, we demonstrated that total CTC number and M-CTC percent could be used to evaluate recurrence risk of HCC patients (Fig. 1e–f). Thus, the recurrence prediction ability of total CTC number and M-CTC percent were explored. Among the 127 HCC patients, the median follow-up time was 38.62 months after hepatectomy (range 1.43–51.13 months), and 42 patients (33.07%) experienced recurrence or distant metastasis as confirmed by imaging test. Total CTC number and M-CTC percent were higher in post-recurrence patients compared to non-recurrence patients (*P* < 0.0001; *P* < 0.0001) (Fig. 2f*,* Table 1). Healthy controls and NMLD patients were also followed for a comparable time, and none developed HCC.

**Fig. 3:**
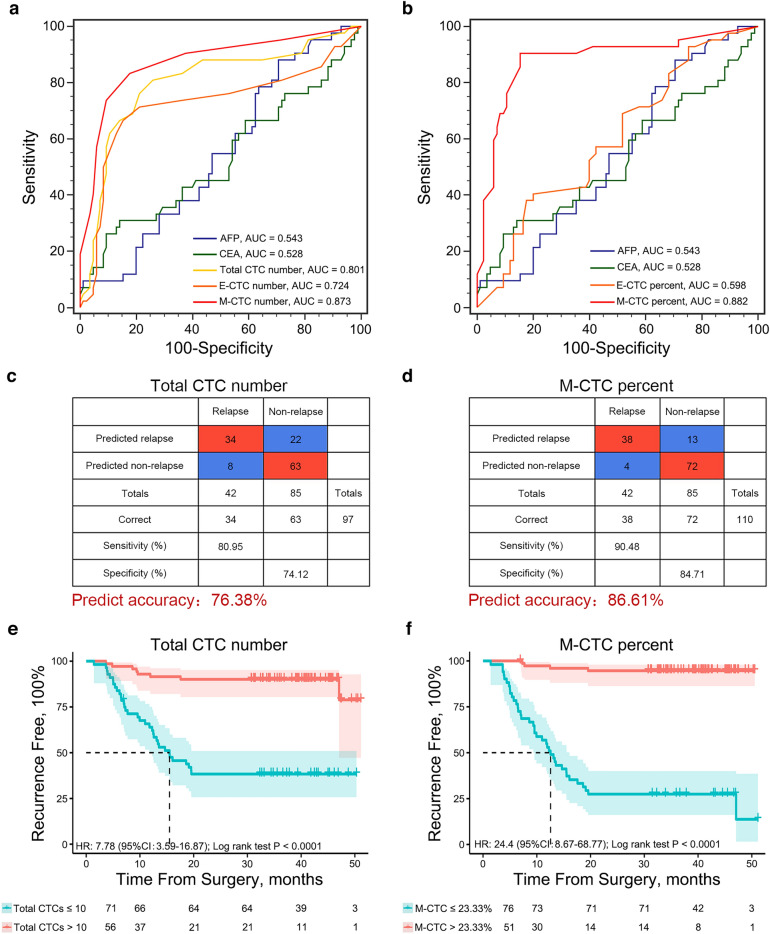
Performance of total CTC number and M-CTC percent in HCC prognosis prediction. **a** The performance of AFP, CEA, E-CTCs, M-CTCs and total CTCs number in HCC recurrence prediction were estimated through ROC curves. **b** The performance of AFP, CEA, E-CTC percent, and M-CTC percent in HCC recurrence prediction were estimated through ROC curves. **c**, **d** Confusion tables of the binary results of total CTC number and M-CTC percent in the HCC cohort. **e**, **f** Kaplan–Meier analysis based on total CTC number and M-CTC percent in predicting recurrence-free survival (RFS) in HCC patients. The cut off value, *P*-values were from Log-rank (Mantel-Cox) tests and HR values are indicated in the figures. The dash lines in the figures represent the median survival (months). *AFP* alpha-fetoprotein, *CEA* carcinoembryonic antigen, *CTCs* circulating tumor cells, *ROC* receiver operating characteristic, *AUC* area under curve, *HR* hazard ratio, *CI* confidence interval

ROC curve analysis was then applied to evaluate the ability of different EMT-related subtype of CTCs and serum tumor biomarker (AFP and CEA) to predict early recurrence/metastasis of HCC. Compared with E-CTC number (AUC = 0.724), the traditional identification subtype of CTCs, the total CTC number (AUC = 0.801) and M-CTC number (AUC = 0.873) both performed better in recurrence prediction, much higher than the AFP (AUC = 0.543) and CEA (AUC = 0.528) (Fig. 3a). Unsurprisingly, the M-CTC percent slightly improved the prognostic ability with an AUC of 0.882 (Fig. 3b). The cut off value used for total CTC number and M-CTC percent were 10/7.5 ml and 23.33%, respectively, which were selected based on the Youden index. Based on this cut off value, both total CTC number and M-CTC percent individually could distinguish 34/42 and 38/42 relapse cases through 2 years follow-up. Additionally, total CTC number and M-CTC percent accurately predicted 63 and 72 of 85 HCC cases without relapse or metastasis. Thus, measuring total CTC number and M-CTC percent yielded sensitivity rates of 80.95% and 90.48%, and specificity rates of 74.12% and 84.71%, respectively, which were significantly higher than when measuring AFP (cut off = 20 ng/ml, sensitivity 59.52%, specificity 44.71%) and CEA (cut off = 5 ng/ml, sensitivity 16.67%, specificity 88.24%) (Fig. 3c, d). Altogether, total CTC number and M-CTC percent had rigid estimate values that were significantly better than those of serum protein AFP and CEA.

### Correlation between the total CTC number and M-CTC percent and the prognosis of HCC patients

Next, Kaplan–Meier analysis was performed to assess the ability of total CTC number and M-CTC percent to predict risk of recurrence. Positive levels of total CTC number (> 10/7.5 ml) and M-CTC percent (> 23.33%) determined by the ROC curve were applied. 34 of the 56 (60.71%) patients positively determined by total CTC number did go through cancer recurrence, while only 8 of the 71 (11.27%) total CTC number-negative patients went through recurrence (Fisher’s exact test, *P* < 0.0001). Similarly, 38 of the 51 (74.51%) M-CTC percent-positive patients experienced HCC recurrence, while only 4 of the 76 (5.26%) M-CTC percent-negative patients (Fisher’s exact test, *P* < 0.0001) had recurrence. The RFS of preoperative total CTC number-positive patients was significantly lower than in total CTC number-negative patients (median RFS for positive and negative groups, 15.5 months vs not reached, log rank *P* < 0.0001; HR 7.78, 95% CI = 3.59–16.87) (Fig. 3e*,* Table 2). Similarly, M-CTC percent level was significantly correlated with worse RFS (12.6 months vs not reached, log rank *P* < 0.0001; HR 24.4, 95% CI = 8.67–68.77) (Fig. 3f*,* Table 2).

**Table 2:** Cox regression model to evaluate recurrence-free survival based on patient characteristics analyzed according to a univariable and multivariable analyses

Variable	Univariable analysis			Multivariable analysis		
	HR	95% CI	*P*	HR	95% CI	*P*
Age (≤ 60 vs > 60)	0.53	0.25–1.11	0.0932			
Sex (male vs female)	1.32	0.61–2.86	0.4749			
HBV infection (negative vs positive)	1.43	0.51–4.00	0.4992			
HCV infection (negative vs positive)	0.90	0.28–2.93	0.8668			
Cirrhosis (yes vs no)	1.31	0.41–4.26	0.6489			
AFP (≤ 20 ng/ml vs > 20 ng/ml)	1.18	0.63–2.19	0.6123			
Tumor diameter (≤ 5 cm vs > 5 cm)	1.40	0.75–2.60	0.2941			
Multiple nodules (yes vs no)	5.60	2.73–11.50	< 0.0001****	3.27	1.47–7.28	0.0036**
MVI (yes vs no)	1.93	1.05–3.57	0.0345*	1.18	0.58–2.39	0.6446
Satellite nodules (yes vs no)	2.01	0.84–4.79	0.1149			
Thrombosis (yes vs no)	1.89	0.93–3.85	0.0803	0.83	0.39–1.78	0.6382
Tumor differentiation (poor and moderated vs well)	2.40	0.33–17.48	0.3869			
AJCC stage (I vs II, III and IV)	3.15	1.51–6.59	0.0023**	0.94	0.39–2.31	0.8974
Total CTC number (≤ 10/7.5 ml vs > 10/7.5 ml)	7.78	3.59–16.87	< 0.0001****	3.49	1.45–8.40	0.0053**
M-CTC percent (≤ 23.33% vs > 23.33%)	24.40	8.66–68.77	< 0.0001****	16.35	5.61–47.67	< 0.0001****

*RFS* recurrence free survival, *HCC* hepatocellular carcinoma, *MVI* microvascular invasion, *AFP* alpha-fetoprotein, *CTC* circulating tumor cell, *HBV* hepatitis B virus, *HCV* hepatitis C virus, *AJCC* American Joint Committee on Cancer

*, **, ***, **** denote a *P* value of < 0.05, < 0.01, < 0.001 and < 0.0001, respectively

Furthermore, univariate and multivariate Cox regression analysis were conducted to investigate the independent risk factors of HCC early recurrence. Based on univariate Cox regression, presence of multiple nodules, MVI, thrombosis, AJCC stage, total CTC number and M-CTC percent were selected for multivariate Cox regression. The analysis revealed that multiple nodules (HR 3.27; 95% CI, 1.47–7.28; *P* = 0.0036), total CTC number (HR 3.49; 95% CI 1.45–8.40; *P* = 0.0053), M-CTC percent (HR 16.35; 95% CI 5.61–47.67; *P* < 0.0001) were independent risk factor for early HCC recurrence (Table 2). These results demonstrated that both total CTC number and M-CTC percent can function as indicating markers of early recurrence of HCC, especially when combined with pathological features, such as multiple nodules.

### CTCs played indispensable role in disease surveillance and relapse alert

**Fig. 4:**
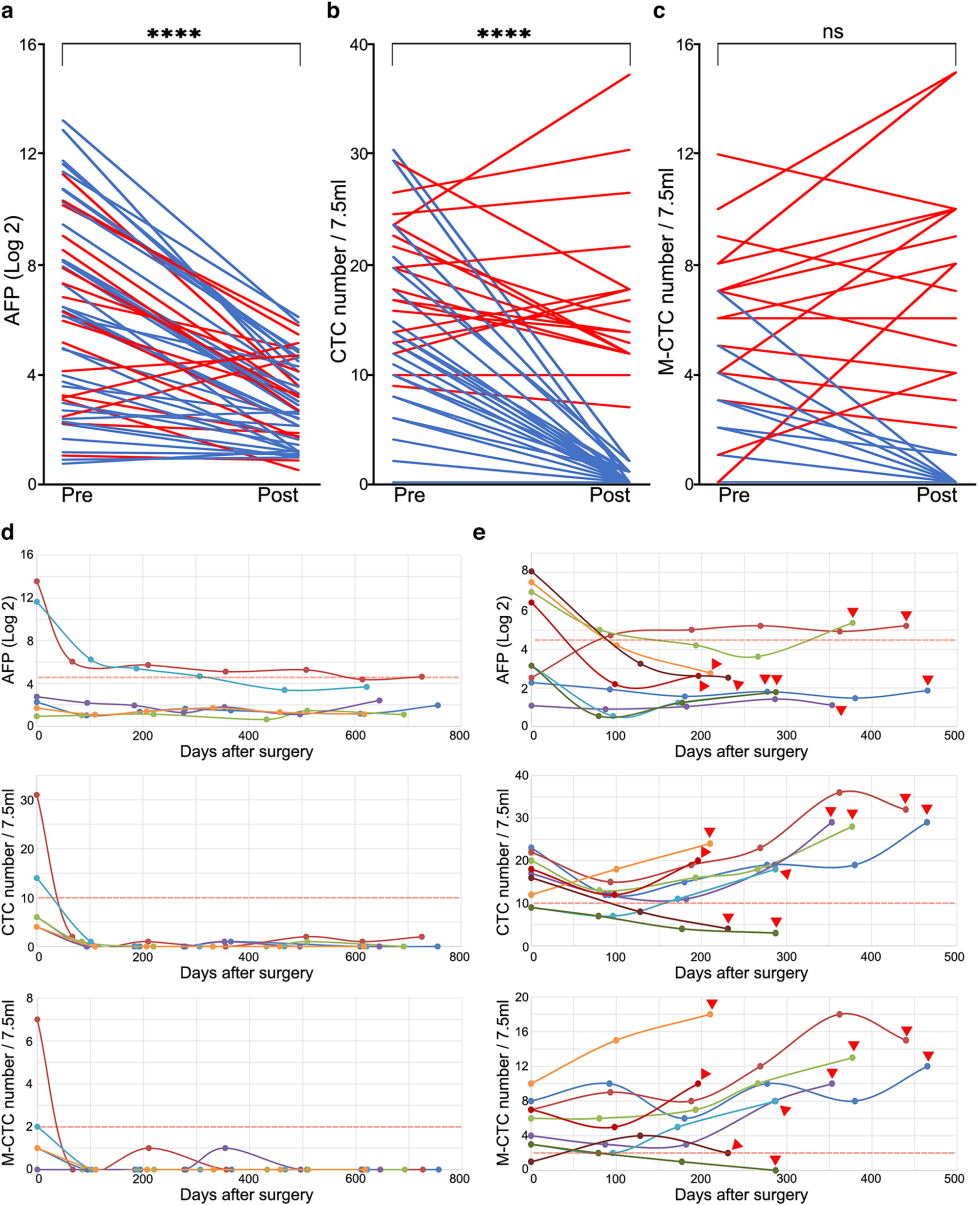
Performance of total CTC and M-CTC number in HCC recurrence supervision. **a**–**c**, The slope plot represented the tendency of AFP (Log2), total CTC number and M-CTC number at the timepoint before and first after surgery (~ 3 month) in follow-up patients. **d**, **e** Preoperative and postoperative serial peripheral blood samples were collected for dynamic monitoring through AFP, total CTC number and M-CTC number in non-recurrence patients (**d**) and recurrence patients (**e**). The red triangles represent recurrence timepoint, the red dash lines represent positive cut off value (AFP > 20 ng/ml, total CTC number > 10/7.5 ml, M-CTC number > 2/7.5 ml)

During follow-up, 52 HCC patients in our cohort were able for the post-operation observation and 18 of them (34.62%) went through the recurrence. We found that reduction in total CTC number and AFP levels from preoperative baseline to the first post-operation timepoint (~ 3 month after surgery) was significantly correlated with the well effect of surgical treatment (*P* < 0.0001 and *P* < 0.0001), while the reverse was occurred in relapse candidates (Fig. 4a–c), especially in M-CTC number. Among patients without progression, 100% (34 of 34) experienced decrease or stable in total CTC number, 100% (34 of 34) in M-CTC number and 91.18% (31 of 34) in AFP from preoperative baseline, while in recurrence patients, 38.89% (7 of 18) saw an ascent in total CTC number from baseline, 61.11% (11 of 18) in M-CTC number from baseline, which was conspicuous higher than 16.67% (3 of 18) of AFP. This performance indicated that M-CTCs, was a more sensitive circuiting tumor marker during longitudinal supervision.

In disease supervision of 15 HCC patients, compared with the basically stable state of 7 non-recurrence cases (Fig. 4d), the changes of CTC number in the recurrence patients were more dramatic. To be specific, total CTC number could alert 7 of 9 (77.78%) recurrence patients. The median interval indicated by CTC number > 10/7.5 ml and recurrence confirmed by MRI/CT/PET image detections was 266 days (range 98–373 days). While M-CTC number (> 2/7.5 ml) could alert 8 of 9 (88.89%) recurrence patients with median interval of 190 days (range 98–373 days). Both were more effective than AFP (> 20 ng/ml), which only valid in 2 of 9 (22.22%) patients with median interval of 322 days (range 297–347 days) (Fig. 4e). For five AFP-negative cases, the dynamic monitoring of CTCs could complement four of them with effective recurrence warning. The results above emphasized indispensability of CTCs, especially M-CTCs dynamic monitoring in early warning of HCC recurrence.

## Discussion

The limitations of measuring serum protein markers in patients with HCC include low sensitivity for early diagnosis, poor prognosis prediction and inability to accurately predict recurrence. AFP is the only serum biomarker currently measured in the clinical management of patients with HCC, but has a 30% false negative rate in HCC diagnosis and even worse performance in relapse prediction (Farinati et al. 2006; Mehta et al. 2017). To address these issues, this study identification the CTCs with different EMT phenotypes: epithelial CTCs (E-CTCs), mesenchymal CTCs (M-CTCs) and CTCs with both epithelial and mesenchymal markers (EM-CTCs) in HCC patients. Then the correlation between tumor malignancy and different subtypes of EMT-related CTCs were explored, and found that total CTC number and M-CTC percent were positively correlated with tumor malignancy parameter. In addition, consistent with previous studies, preoperative total CTC number and M-CTC performed better than that of AFP in HCC recurrence prediction (Court et al. 2018; Qi et al. 2018). As for the longitudinal supervision of HCC patients, we found that CTCs, especially M-CTCs were indispensable of in early warning of HCC recurrence.

Due to their low abundance in the peripheral blood, the main technical difficulty of CTC-analysis is the lack of an effective enrichment and identification system. Previous detection methods were largely based on expression of epithelial markers, such as EpCAM and CK, which are widely expressed in solid tumors of epithelial origin. For example, the CellSearch system approved by the Food and Drug Administration for CTCs detection in the identification of metastatic breast cancer, prostate cancer and colon cancer is based on the EpCAM expression (Riethdorf et al. 2007). While regarded as the stem-like cancer cell, EpCAM-positive cells account for only 30% of HCC, thus making the CellSearch system unsuitable for CTCs detection in HCC (Ruck et al. 2000; Shen et al. 2018). In this study, our negative enrichment strategy was based on magnetic activated cell sorting (MACS) to remove the leukocytes, enriching for CTCs in the peripheral blood of HCC. The subsequent phenotype identification was performed using immunofluorescence staining of different markers. This strategy has good potential for application in the clinic due to ease of operation and minimal dependency on difficult-to-access equipment.

CTCs are highly heterogeneous, EMT-like CTCs have been demonstrated to be the possible contributors to distant metastases and tumor progression in solid tumors (Dong et al. 2020; Gao et al. 2021). EMT gives primary tumor cells the ability to break through the extracellular matrix binding, invade into the circulatory system and form distant metastases (Shook and Keller 2003). During this process, expression of epithelial markers such as EpCAM and CK decrease, while expression of mesenchymal markers rise, leading to false negatives in CTCs identification (Mikolajczyk et al. 2011). CTCs undergoing EMT share characteristics with cancer stem cells, including stronger invasive, metastasic abilities, higher tolerance to chemotherapy and targeted therapeutic drugs (Mitra et al. 2015). However, due to the lack of specific marker molecules and detection methods for EMT-like CTCs, relevant studies are limited.

Vimentin is an important component of intermediate filaments that is extensively expressed in mesenchymal cells. It is also overexpressed in breast cancer, cancers of the central nervous system, prostate cancer, malignant melanoma and lung cancer and is measured in the detection step of the CellSearch system (Satelli and Li 2011). Recent studies have found that glioblastoma cells expressing vimentin have strong proliferative ability in vitro (Noh et al. 2016). Therefore, vimentin could also be used as a broad-spectrum marker for CTC-detection, as patients with positive vimentin have worse response to treatment (Satelli et al. 2015, 2014).

In this study, vimentin was used to detect M-CTCs, and the epithelial marker CK was used to detect E-CTCs. Using these methods, we found a high heterogeneity of CTCs in the peripheral blood of patients with HCC. Furthermore, we evaluated the efficacy of the total CTC number and M-CTC percent in prognosis prediction in a large HCC cohort, and found that these markers outperformed than the serum marker AFP for early diagnosis and prognosis prediction. M-CTC percent were more effective in prognosis progression compared with total CTC number, suggesting that vimentin positive CTCs may contribute to worse outcomes. In addition, high CTC number and M-CTC percent were significantly associated with poor RFS rate in HCC patients and worked as independently significant risk factors for HCC early recurrence. We also evaluated the supervision ability of total CTC and M-CTC in longitudinal HCC patients, both worked more effectively than AFP in warning rate and lead time. Inevitably, there are some shortcomings of our research: (1) only EMT-related subtypes of CTCs were measured, which may neglect the complexity given by the full heterogeneity of CTCs; (2) The number of cases used for survival analysis is relatively small, and related conclusions require further verification in a large independent cohort. In brief, our study supports the application of CTCs as more effective and accurate than those serum biomarkers currently used in the clinic in HCC prognosis prediction and supervision, which provide a foundation for future studies.

## Supplementary Information

Below is the link to the electronic supplementary material.

Supplementary file1 (DOC 85 kb)

## Data Availability

Data are available in a public repository. All data relevant to the study are included in the article or uploaded as online supplemental information. Additional data related to this paper may be requested from the corresponding authors.
