# Supplementary material for: The mesenchymal circulating tumor cells as biomarker for prognosis prediction and supervision in hepatocellular carcinoma
Source: J Cancer Res Clin Oncol. 2023 Jan 12;149(9):6035–48. doi: 10.1007/s00432-022-04526-9 (PMC10356895; doi:10.1007/s00432-022-04526-9)
Supplement: Supplementary file 1 — Supplementary file1 (DOC 85 kb) [file 432_2022_4526_MOESM1_ESM.doc]

**Table S1. Clinical and Pathological Characteristics of HCC Patients**

| **Clinicopathologic parameters** | | |
| --- | --- | --- |
| Age (*N* = 127) | Median years 55 (28-77) | |
| Sex (*N* = 127) | Female | 21 (16.53%) |
|  | Male | 106 (83.46%) |
| ALT (U/L) (*N* = 127) | Median (ng/ml) 39 (10–3053) | |
| AST (U/L) (*N* = 127) | Median (ng/ml) 43 (13–2280) | |
| TBIL (umol/L) (N = 127) | Median (ng/ml) 15.5 (4.3–80.7) | |
| ALP(U/L) (N = 127) | Median (ng/ml) 65 (35.5–678) | |
| AFP (ng/ml) (*N* = 127) | Median (ng/ml) 33.78 (1.01–131344) | |
| CEA (ng/ml) (*N* = 127) | Median (ng/ml) 2.475 (0.62–112.2) | |
| Largest tumor diameter (mm) (*N* = 127) | Median (mm) 40 (12–200) | |
| Multifocal (*N* = 127) | Absent | 112 (88.19%) |
|  | Present | 15 (11.81%) |
| HBV (*N* = 127) | Absent | 16 (12.60%) |
|  | Present | 111 (87.40%) |
| HCV (*N* = 127) | Absent | 116 (91.34%) |
|  | Present | 11 (8.66%) |
| Cirrhosis (*N* = 127) | Absent | 11 (8.66%) |
|  | Present | 116 (91.34%) |
| Prior treatment (*N* = 127) | Absent | 88 (69.29%) |
|  | Present | 39 (30.71%) |
| Macrovascular invasion (*N* = 127) | Absent | 73 (57.48%) |
|  | Present | 54 (42.52%) |
| Tumor thrombosis (*N* = 127) | Absent | 107 (84.25%) |
|  | Present | 20 (15.75%) |
| Differentiation grade (*N* = 127) | Well | 4 (3.15%) |
|  | Well-Moderately | 2 (1.57%) |
|  | Moderately | 79 (62.20%) |
|  | Moderately-Poorly | 19 (14.96%) |
|  | Poorly | 23 (18.11%) |
| Neoadjuvant (*N* = 39) | Radiofrequency ablation | 17 (43.59%) |
|  | Transcatheter arterial chemoembolization | 14 (35.90%) |
|  | Radiotherapy | 8 (20.51%) |
| AJCC stage (*N* = 126) | Ι | 53 (42.06%) |
|  | II | 46 (36.51%) |
|  | III | 26 (20.63%) |
|  | IV | 1 (0.79%) |
| Child Pugh class (*N* = 127) | A | 118 (92.91%) |
|  | B | 8 (6.30%) |
|  | C | 1 (0.78%) |
| BCLC stage (*N* = 127) | A | 85 (66.93%) |
|  | B | 14 (11.02%) |
|  | C | 28 (22.05%) |
|  | D | 0 (0.00%) |
| AFP, alphafetoprotein; HBV, hepatitis B virus; HCV, hepatitis C virus; AJCC, American Joint Committee on Cancer; BCLC, Barcelona clinic liver cancer stage; MVI, microvascular invasion; ALT: Alaninetransaminase; AST: Alaninetransaminase; TBIL: total bilirubin; ALP: alkaline phosphatase; CEA, Carcinoembryonic antigen. | | |

**Table S2. Clinical characteristics of healthy volunteer**

| **Clinicopathologic parameters** | | |
| --- | --- | --- |
| Age (*N* = 42) | Median years 54 (23-76) | |
| Gender (*N* = 42) | Female | 13 (30.95%) |
|  | Male | 29 (69.05%) |
| AFP (ng/ml) (*N* = 42) | Median (ng/ml) 3.09 (1.31–42.68) | |
| CEA (ng/ml) (*N* = 42) | Median (ng/ml) 2.44 (1.05–36.82) | |
| HBV (*N* = 42) | Absent | 41 (97.62%) |
|  | Present | 1 (2.38%) |
| HCV (*N* = 42) | Absent | 42 (100.0%) |
|  | Present | 0 (0.00%) |
| Cirrhosis (*N* = 42) | Absent | 41 (97.62%) |
|  | Present | 1 (2.38%) |
| E-CTCs (*N* = 42) | Median number/7.5ml 0 (0-1.5) | |
| EM-CTCs (*N* = 42) | Median number/7.5ml 0 (0-1.0) | |
| M-CTCs (*N* = 42) | Median number/7.5ml 0 (0-0.5) | |
| AFP, alphafetoprotein; HBV, hepatitis B virus; HCV, hepatitis C virus; E-CTCs, epithelial CTCs; EM-CTCs, epithelial-mesenchymal CTCs; M-CTCs, mesenchymal CTCs. | | |

**Table S3. Clinical characteristics of nonmalignant liver disease (NMLD) patients**

| **Clinicopathologic parameters** | | |
| --- | --- | --- |
| Age (*N* = 21) | Median years 52 (32-72) | |
| Gender (*N* = 21) | Female | 3 (14.29%) |
|  | Male | 18 (85.71%) |
| AFP (ng/ml) (*N* = 21) | Median (ng/ml) 4.26 (1.67–228.72) | |
| CEA (ng/ml) (*N* = 21) | Median (ng/ml) 2.48 (1.01–12.48) | |
| HBV (*N* = 21) | Absent | 2 (9.52%) |
|  | Present | 19 (90.48%) |
| HCV (*N* = 21) | Absent | 20 (95.24%) |
|  | Present | 1 (4.76%) |
| Type (*N* = 21) | Cirrhosis | 15 (71.43%) |
|  | Hepatic hemangioma | 3 (14.29%) |
|  | liver adenoma | 1 (4.76%) |
|  | focal nodular hyperplasia | 2 (9.52%) |
| Cirrhosis (*N* = 21) | Absent | 5 (23.81%) |
|  | Present | 16 (76.19%) |
| E-CTCs (*N* = 21) | Median number/7.5ml 0 (0-8.0) | |
| EM-CTCs (*N* = 21) | Median number/7.5ml 0 (0-1.5) | |
| M-CTCs (*N* = 21) | Median number/7.5ml 0 (0-1.0) | |
| AFP, alphafetoprotein; HBV, hepatitis B virus; HCV, hepatitis C virus; E-CTCs, epithelial CTCs; EM-CTCs, epithelial-mesenchymal CTCs; M-CTCs, mesenchymal CTCs. | | |
